# Effectiveness of bystander cardiopulmonary resuscitation in improving the survival and neurological recovery of patients with out-of-hospital cardiac arrest: A nationwide patient cohort study

**DOI:** 10.1371/journal.pone.0243757

**Published:** 2020-12-16

**Authors:** Joongyub Lee, Woojoo Lee, Yu Jin Lee, Hyunman Sim, Won Kyung Lee

**Affiliations:** 1 Department of Preventive Medicine, Seoul National University College of Medicine, Seoul, South Korea; 2 Department of Public Health Science, Graduate School of Public Health, Seoul National University, Seoul, South Korea; 3 Department of Emergency Medicine, Inha University Hospital, School of Medicine, Inha University, Incheon, South Korea; 4 Department of Prevention and Management, Inha University Hospital, School of Medicine, Inha University, Incheon, South Korea; St. Michael's Hospital, CANADA

## Abstract

**Introduction:**

Few studies have focused on enhancing causality and yielding unbiased estimates on the effectiveness of bystander cardiopulmonary resuscitation (BCPR) on the outcomes of out-of-hospital cardiac arrest (OHCA) in a real-world setting. Therefore, this study evaluated the effect of BCPR on the outcomes of OHCA and its differences according to the characteristics of OHCA.

**Methods:**

This study enrolled all patients with OHCA of cardiac etiology treated by emergency medical services (EMS) in Korea from 2012 to 2015. The endpoints were survival and neurological recovery at discharge, and the main exposure was BCPR conducted by a layperson. The effect of BCPR was analyzed after adjusting for confounders, determined using a directed acyclic graph, by inverse probability of treatment weighting (IPTW) and model-based standardization (STR). Moreover, differences in subgroups and time trends were evaluated.

**Results:**

Among 10,505 eligible patients after excluding those with missing data on BCPR, 7,721 patients received BCPR, accounting for 74.3% of EMS-treated OHCA patients. BCPR increased the odds of survival and good neurological recovery at discharge by 1.67- (95% confidence interval (CI): 1.44–1.93) and 1.93- (95% CI: 1.56–2.39) fold, respectively, in the IPTW analysis. These findings were comparable to those obtained with STR. The odds ratios were 2.39 (95% CI: 1.91–2.94) and 2.70 (95% CI: 1.94–3.41), respectively, in the sensitivity analysis of the missing BCPR information considering confounders and the outcome variable. However, the effect of qualified BCPR was not evenly distributed, and it did not increase with time. BCPR was likely to be more effective in male patients aged <65 years, those who experienced an OHCA in a private place or non-capital region, and those with shockable rhythm at the scene.

**Conclusion:**

Based on data from a nationwide registry, the estimated effect of BCPR on survival and neurological recovery was moderate and did not improve from 2012 to 2015.

## Introduction

Out-of-hospital cardiac arrest (OHCA) is a leading cause of death and a major public health problem. According to the American Heart Association, more than 356,000 OHCA cases are recorded annually in the US, while 275,000 OHCA cases are recorded annually in Europe [1, 2]. Globally, the overall incidence is approximately 95.9 per 100,000 person-years [3]. Although the survival rate on hospital discharge of patients with emergency medical services (EMS)-treated OHCA increased from 10.2% in 2006 to 12.4% in 2015, nearly 90% of cases are fatal [1]. In addition to mortality, cardiac arrest has a considerable economic burden due to profound neurologic disability. The societal burden of OHCA is comparable to or even greater than that of other leading causes of death [4, 5]. A previous study showed that the burden of nontraumatic OHCA in the US was approximately 1,347 disability-adjusted life years per 100,000 population in 2016, ranking third after ischemic heart disease (2,447) and low back and neck pain (1,565) [6].

Early recognition and treatment of OHCA are likely to increase resuscitation’s success rate and improve neurological outcomes. Hence, bystander cardiopulmonary resuscitation (BCPR) is associated with increased survival and better neurologic outcomes in patients with OHCA. Due to the dissemination of information about BCPR, the rate of layperson-initiated CPR in OHCA cases increased from 36.5% in 2006 to 40.7% in 2016 [1]. However, the delivery of BCPR differs across subgroups. A recent article showed that the provision of BCPR was associated with the socioeconomic status of the area in which the OHCA occurred [7]. Another review reported gender disparities in the provision of BCPR: women were more likely to receive BCPR and had increased odds of survival after sudden cardiac arrest, although they were less likely to have witnessed OHCA [8]. However, a study based on the Resuscitation Outcomes Consortium registry in the US reported that men had a higher likelihood of receiving BCPR and having improved survival than women [9]. In addition to BCPR provision, the effect of BCPR on survival and neurologic outcomes may vary across subgroups. Previous studies have suggested that the effect of BCPR on the outcomes of OHCA could be modified by the patient’s age, type of bystander, and place of OHCA; however, the most important determinant was the quality of BCPR [10].

Limited research has focused on enhancing causality and yielding unbiased estimates on the effectiveness of BCPR in improving OHCA outcomes in a real-world setting [11]. The odds ratio for an increase in survival varied remarkably, from 1.03 to 5.1, which could be explained by different distributions of confounders or effect modifiers [10]. Previous studies had different sets of confounders because they did not use directed acyclic graphs (DAGs) to define the confounders of the effect of BCPR on OHCA outcomes, which would have allowed the systematic inclusion of confounders [12]. Moreover, these previous studies did not use statistical tools such as standardization and inverse probability of treatment weighting (IPTW), which would have helped mimic the situation of an ideally randomized experiment in terms of causal inference to yield unbiased estimates of the effects of BCPR [13]. These methods can be used to consider the interactions between confounders, which would reflect the complex relationships between confounders in the real world when estimating the effects of BCPR on the outcomes of OHCA. Therefore, this study applied DAGs and appropriate statistical methods to mimic randomization to evaluate the effectiveness of BCPR in increasing survival and neurological recovery.

This study aimed to estimate the effect of BCPR on the survival and neurologic outcomes at the discharge of OHCA patients, based on confounders identified using a DAG, and determine the differences in this effect based on the patients’ characteristics and OHCA.

## Materials and methods

### Study population

We used data from the Korea Out-of-Hospital Cardiac Arrest Registry (KOHCAR), a national registry established by the Korea Centers for Disease Control and Prevention (KCDC) that contains information about OHCA from all 712 emergency departments (EDs) in Korea. Medical record review experts were employed and trained by the KCDC to review variables related to OHCA risk and outcomes based on the Utstein guidelines [14]. They were dispatched to all EDs to gather information on hospital care and patients’ outcomes with OHCA. A data quality management committee comprising emergency physicians, epidemiologists, statistical experts, and medical record review experts oversaw data collection and provided feedback to ensure the quality of the medical record review process, with monthly reviews of the collected data [15]. The KOHCAR was developed based on recommendations from the international OHCA database. The variables collected by the KCDC were based on the Utstein 2004 template. These included age, sex, place of the event, witness status, bystander CPR, bystander defibrillation, cause of cardiac arrest (cardiac, trauma, poisoning, drowning, asphyxia/hanging, and other), initial electrocardiography (ECG) rhythm (ventricular fibrillation [VF]/pulseless ventricular tachycardia [VT], pulseless electrical activity [PEA], asystole), date and time of onset (season, weekday/weekend, and day/night), EMS defibrillation, achievement of prehospital return of spontaneous circulation (ROSC), survival to discharge, and a measure of neurological recovery. The database contained anonymized and de-identified information about OHCA and could be accessed with KCDC authorization. However, data on the variables in the EMS CPR registry and EMS run sheet collected by the Central Fire Services are not accessible to the public.

The full details of the KOHCAR goals and protocols were published previously [15]. This study was approved by the Institutional Review Board of Inha University Hospital (INHAUH2018-11-006), which waived the need for informed consent. The following inclusion criteria were applied to all OHCA patients in the KOHCAR: 1) age 18 years and older, 2) presumed cardiac etiology, and 3) occurrence of OHCA from 2012 to 2015. Patients with OHCA witnessed by medical personnel and those with unknown information about BCPR were excluded.

### BCPR training program in Korea

The national CPR training program was first launched and has been promoted since the early 2000s by the KCDC. The EMS division of the Ministry of Health and Welfare financially supports 16 provincial governments to conduct CPR campaigns and provide CPR courses. In 2012, 223,952 CPR trainees received government support [16]. Mandatory training programs for legally defined first responders, such as drivers, schoolteachers, police officers, rescuers, and guards, were started under the EMS Act of 2008 [15]. Another obligatory training program for students and teachers was implemented in 2012 under the School Health Act. All students in each primary, middle, and high school were required to attend at least one CPR training session during each school year. Moreover, each provincial government sets up training sites and conducts its program according to the standard recommended by the KCDC. In addition, hospitals, nongovernmental organizations, academic and scientific societies, and fire departments also provide CPR training. The public-access defibrillator program was approved by the National Assembly in 2008, and the first automated external defibrillators (AEDs) were installed in 2010 [17]. However, only a small number of AEDs are currently deployed in public places and were rarely used by bystanders in the current study.

### Korean EMS system

The Korean EMS system is a basic-to-intermediate level ambulance service operated by 16 provincial headquarters of the National Fire Department and a single-tiered, fire-based EMS system [18]. The most qualified emergency medical technician (EMT) can perform CPR with an AED, evaluate the cardiac rhythm on-site, provide advanced airway management, and administer intravenous fluids. EMTs cannot declare death or stop CPR on the scene unless the patient regains a pulse in the field or during transport to an ED; therefore, all EMS-assessed patients are transported to the nearest ED. The reported mean ± standard deviation of the response time for OHCA, defined as the time interval from an emergency call to ambulance arrival at the scene, was 6.9 ±3.3 min [19]. Moreover, the mean prehospital time (time from an emergency call to ED arrival) was 21.8 ± 8.4 min in 2009–2011.

### Main outcomes

The primary outcomes were survival and a good neurologic outcome at hospital discharge. Good neurological recovery was defined as a cerebral performance category score of 1 (good cerebral performance) or 2 (moderate cerebral disability) [20].

### Variables and measurements

The main exposure was BCPR, defined as CPR administered by a layperson or first responder who was not an EMT or medical personnel, based on the ED medical records. Potential confounders were selected on the basis of the DAG (S1 Fig). The first responders in this analysis included police officers, school teachers, security guards at sports facilities and tourist attraction sites, and rescuers [15].

The confounders included in the main analysis were patient demographic characteristics (age, sex, and health insurance type [reflecting socioeconomic status]) and EMS factors (public/private place, presence of a witness, region, and year). The region was categorized as a metropolitan city or other; a metropolitan city was defined as an administrative district with a population of 1,000,000 or more. There were no missing values for age, sex, witness, region, and year among the confounders. When medical record reviewers could not find information on the place of OHCA and the insurance type of OHCA patients, they were categorized as unknown place and unknown insurance type and then included in the main analysis and sensitivity analysis considering the missing BCPR data. The proportions of unknown values on the place of OHCA and the insurance type of OHCA patients were 14.2% and 6.5%, respectively, among 40,888 patients in the sensitivity analysis.

The primary ECG rhythm at the scene was considered an intermediate variable between BCPR and the outcomes (survival to discharge and neurological recovery). Therefore, the final model was not adjusted for primary ECG rhythm (either shockable or non-shockable).

### Statistical analysis

Categorical variables are presented as counts and percentages and continuous variables as means and standard deviations in the descriptive analysis. Chi-square and Student’s t-tests were used to compare characteristics between the groups with and without BCPR. The confounders were selected after a review of the related literature and were finalized using a DAG, which is a graphical representation of the causal relationships of variables that provide testable implications such as conditional independence [17, 21].

Two statistical methods to control for confounding bias were applied to estimate the effect of BCPR on the outcomes of OHCA patients. IPTW was applied using propensity scores, and the results were compared with those obtained via model-based standardization (STR) to ensure the robustness of the analytic methods. These methods are commonly used in observational studies to control for confounding and estimate the causal parameters of interest [13].

A sensitivity analysis was conducted using combinations of confounders identified using the DAG. Each combination was supposed to block all “back-door paths” from BCPR to survival or neurologic recovery at discharge. We also conducted sensitivity analyses considering all two-way interactions between the covariates in the model to evaluate the robustness of the effects of BCPR on OHCA outcomes while considering all interactions. Moreover, we estimated the time trends and differences in the effects of BCPR on the outcomes of OHCA among the subgroups.

Since the proportion of patients with missing BCPR data was high, additional sensitivity analyses were conducted to assess the changes in the effects of BCPR on the outcomes of OHCA patients. We calculated odds ratios under the missing-at-random assumption using a modified weight in the IPTW analysis [22]. The modified weight was determined by multiplying the propensity score or 1-propensity score and the probability that the BCPR status was observed. In modeling the missing probability, we considered two cases: the missing probability depended on (1) only confounders and (2) both confounders and the outcome variable.

A standardized difference was considered statistically significant if its absolute value was >0.1. The other analyses were performed on two sides, and a p <0.05 was considered statistically significant. All statistical analyses were performed using R version 4.0.0 (R Project, Vienna, Austria).

## Results

**Fig 1:**
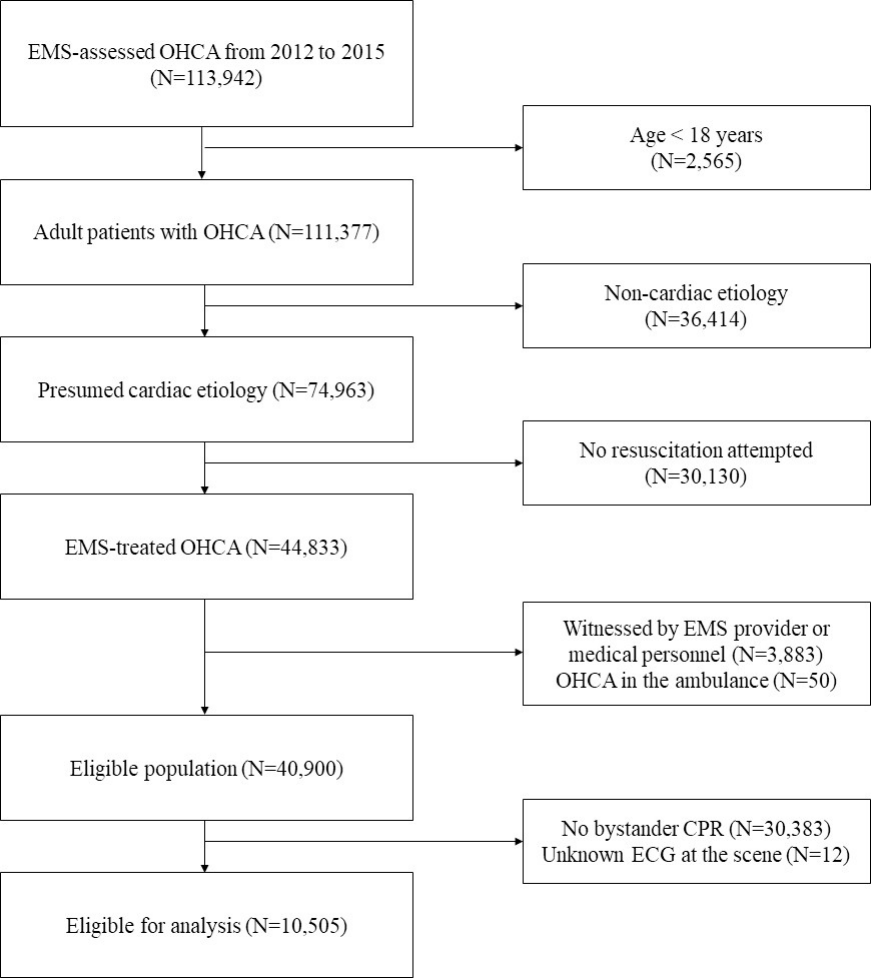
Flowchart of study inclusion. Abbreviations: OHCA, out-of-hospital cardiac arrest; EMS, emergency medical service; CPR, cardiopulmonary resuscitation; ECG, electrocardiogram.

Of the 113,942 patients with EMS-assessed OHCA from 2012 to 2015, 40,900 met the inclusion criteria. However, 74.3% of the 40,900 patients with OHCA had missing BCPR data. After excluding patients with missing BCPR data in the ED records, 10,505 patients were eligible for the final analysis. Additionally, patients with missing BCPR data were further evaluated in sensitivity analyses. The process of applying the inclusion and exclusion criteria is summarized in Fig 1.

The demographic characteristics, according to BCPR provision, are presented in Table 1. In total, 7,721 patients received BCPR, while 2,784 did not. The patients who received BCPR were younger than those who did not (64.7 vs. 67.5 years, p <0.001). The proportion of male participants was higher than that of female participants in both the BCPR and no-BCPR groups, although the difference was not statistically significant (p = 0.065). Moreover, OHCA events involving BCPR were more likely to occur in a public place (24.3%) than events not involving BCPR (19.9%). OHCA occurred more commonly in metropolitan cities in both the BCPR and no-BCPR groups, while the proportion of events in metropolitan cities was higher in the no-BCPR group than in the BCPR group. In addition, patients who received BCPR were more likely to have experienced a witnessed OHCA than those who did not receive BCPR (59.5% vs. 49.4%), and BCPR was more likely to be correlated with a primary shockable rhythm than with a primary non-shockable rhythm at the scene (24.4% vs. 15.7%). In 70 patients with OHCA, shock was delivered by a layperson using a public AED. The BCPR group had a higher proportion of patients who experienced ROSC before ED arrival, survived to discharge, and had good neurological recovery than the no-BCPR group. A total of 2,328 and 590 patients in the BCPR and no-BCPR groups, respectively, were discharged from the ED and survived to admission (data not shown). After applying the IPTW method, the BCPR and no-BCPR groups became balanced with the absolute value of standardized difference <0.1 in the baseline characteristics (S1 Table and S3 Fig).

**Table 1:** Characteristics of the study population.

	Total	BCPR	No BCPR	p-value
	N = 10,505	N = 7,721	N = 2,784	
**Confounders**				
Age (years), mean (SD)	65.4±16.0	64.7±16.1	67.5±15.3	<0.001
Sex, %				
Male	7,197 (68.5)	5,329 (69.2)	1,868 (67.1)	0.065
Place, %				
Public	2,428 (23.1)	1,874 (24.3)	554 (19.9)	<0.001
Non-public	6,905(65.7)	4,872(63.1)	2,033(73.0)	
Unknown	1,172 (11.2)	975 (12.6)	197 (7.1)	
Region, %				
Metropolitan cities	5,911 (56.3)	4,145 (53.7)	1,766 (63.4)	<0.001
Presence of a witness, %				
Witnessed	5,973 (56.9)	4,597 (59.5)	1,376 (49.4)	<0.001
Year, %				
2012	1,796 (17.1)	1,062 (13.8)	734 (26.4)	0.006
2013	2,150 (20.5)	1,517 (19.7)	633 (22.7)	
2014	3,161 (30.1)	2,370 (30.7)	791 (28.4)	
2015	3,398 (32.4)	2,772 (35.9)	626 (22.5)	
Type of insurance, %				
NHI	9,275 (88.3)	6,849 (88.7)	2,426 (87.1)	0.015
Medical aid	678 (6.5)	462 (6.0)	216 (7.8)	
Unknown	552 (5.2)	410 (5.3)	142 (5.1)	
**Outcomes**				
Primary shockable rhythm	2,321(22.1)	1,885(24.4)	436(15.7)	<0.001
at the scene, %				
ROSC before ED arrival, %	1,185 (11.3)	1,065 (13.8)	120 (4.3)	<0.001
Survival to discharge, %	1,930 (18.4)	1,627 (21.1)	303 (10.9)	<0.001
Good neurological recovery, %	989 (9.9)	868 (11.9)	121 (4.5)	<0.001

BCPR, bystander cardiopulmonary resuscitation; ROSC, return of spontaneous circulation; ED, emergency department; NHI, National Health Insurance; Primary rhythm, Primary rhythm at the scene.

BCPR increased the odds of survival to hospital discharge by 1.67-fold (95% confidence interval (CI): 1.44–1.93) in IPTW analysis, considering all two-way interactions between confounders (Table 2). The odds ratio was 1.68 (95% CI: 1.45–1.94) when the interactions between confounders were not considered. The odds ratios were 1.93 (95% CI: 1.56–2.39) and 1.97 (95% CI: 1.59–2.45), respectively, in terms of good neurological recovery. If sex was not considered, the odds ratios of BCPR were 1.66 (95% CI: 1.44–1.91) and 1.91 (95% CI: 1.54–2.37) for survival and neurological recovery at discharge, respectively. Moreover, patients who received BCPR had a 1.89-fold (95% CI: 1.64–2.17), and 2.34-fold (95% CI: 1.90–2.89) increased odds of survival to discharge and good neurologic recovery, respectively, when age was not considered. In addition, the effects of BCPR on survival to discharge and neurological outcomes in the IPTW analysis were similar to those observed in the STR (S2 Table).

**Table 2:** Odds ratios of bystander cardiopulmonary resuscitation for survival and neurologic outcomes at discharge.

Covariates ^a^		Survival to discharge	Neurological recovery
Set 1^a^	Interaction^b^	1.67 (1.44–1.93)	1.93 (1.56–2.39)
	No interaction	1.68 (1.45–1.94)	1.97 (1.59–2.45)
Set 2	Interaction^b^	1.66 (1.44–1.91)	1.91 (1.54–2.37)
	No interaction	1.68 (1.45–1.94)	1.97 (1.59–2.45)
Set 3	Interaction^b^	1.89 (1.64–2.17)	2.34 (1.90–2.89)
	No interaction	1.84 (1.60–2.12)	2.27 (1.84–2.80)

^a^ Variables in the directed acyclic graph were used as covariates in the model.

Set 1: (Place, Insurance, Region, Witness, Year, Sex, Age);

Set 2: (Place, Insurance, Region, Witness, Year, Age);

Set 3: (Place, Insurance, Region, Witness, Year, Sex).

^b^ Interaction: all two-way interaction terms among the covariates are included.

**Fig 2:**
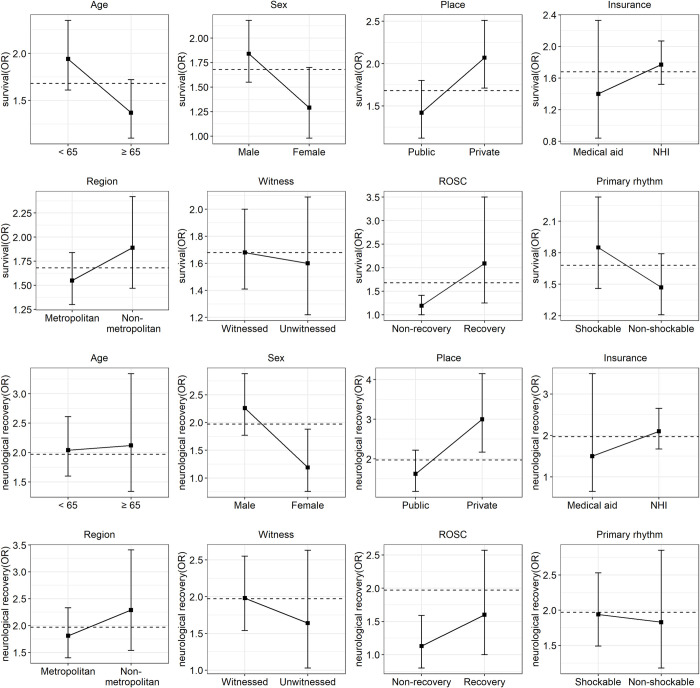
Odds ratios of bystander cardiopulmonary resuscitation (BCPR) for survival and neurological recovery at discharge using inverse probability of treatment weighting according to the characteristics of patients with out-of-hospital cardiac arrest (OHCA).

The influence of BCPR on recovery depended on the patients' characteristics or events (Fig 2). Male patients and those aged <65 years were more likely to survive to discharge than female patients and those aged >65 years. Moreover, patients who experienced OHCA in a private place were more likely to show survival to discharge than those who experienced OHCA in a public place. Similar patterns were observed for the model established for neurological recovery. However, the result was only statistically significant for sex when STR was applied (S3 Table). BCPR was also effective in patients with a primary non-shockable rhythm and those without ROSC before ED arrival. These findings were robust even when STR was used for assessing causal inference (S3 Fig).

**Fig 3:**
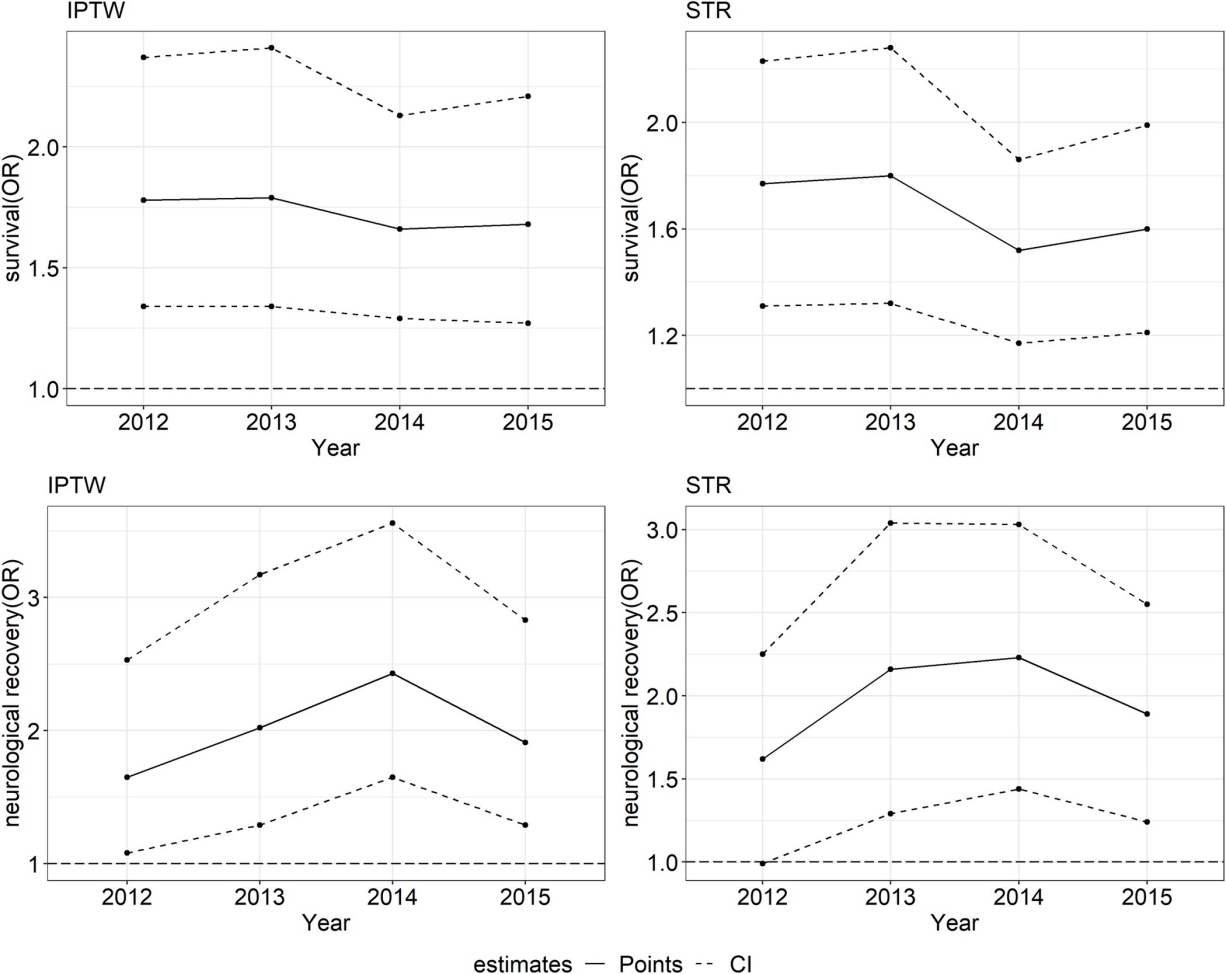
Time trends in the effects of bystander cardiopulmonary resuscitation (BCPR) on survival to hospital discharge and neurological recovery, 2012–2015. IPTW, inverse probability of treatment weighting; STR, model-based standardization.

No continuous increase or decrease was observed in the trend of the influence of BCPR (Fig 3). The odds ratio of BCPR for survival at discharge ranged from 1.66 to 1.79 in 2012–2015 in the IPTW analysis, and this finding was comparable to that obtained with STR. In the IPTW analysis, the odds ratio of BCPR for good neurological recovery changed from 1.65 (95% CI: 1.08–2.53) in 2012 to 2.43 (95% CI: 1.65–3.56) in 2014 and then returned to 1.91 (95% CI: 1.29–2.83) in 2015. Moreover, the 95% CIs overlapped during the study period for survival and neurological recovery at discharge.

OHCA patients who received BCPR were more likely to survive hospital discharge and show good neurological recovery even when the missing BCPR values were considered. The odds ratios for survival to discharge changed from 1.68 (95% CI: 1.45–1.94) to 2.39 (95% CI: 1.91–2.94); however, the CI was overlapped when the probability was estimated using both confounders and the outcome (S4 Fig). Regarding good neurological recovery, the value changed from 1.97 (95% CI: 1.59–2.45) to 2.70 (95% CI: 1.94–3.41) when the probability was estimated using both confounders and the outcome. Furthermore, the odds ratios for survival to hospital discharge and good neurological recovery showed similar patterns when sex or age was not included in the model. The adjusted odds ratios (AORs) hardly changed if the probability of missing values was estimated using only the confounders. The changes observed were as follows: 1.68 (95% CI: 1.45–1.94) to 1.68 (95% CI: 1.45–1.96) for survival to discharge and 1.97 (95% CI: 1.59–2.45) to 1.96 (95% CI: 1.55–2.47) for good neurological recovery, if the probability of missing values was estimated using only confounders.

The validity of our findings was evaluated using three models with more complicated direct and indirect links (S5 Fig). First, we added potential direct links between sex and outcome, between place and BCPR, and between place and outcome to the DAG. The confounder set that we needed to adjust was (Insurance, Region, Witness, Year, Sex, Place). Second, we added potential direct links between sex and outcome, between place and BCPR, and a link between age and witness status to the DAG. The confounder sets that we needed to consider were (Insurance, Region, Witness, Year, Sex, Age) or (Insurance, Region, Witness, Year, Sex, Place). Third, we added potential direct links between sex and outcome and between place and BCPR, a direct link between place and outcome, and links between age and witness status and between insurance and metropolitan area to the DAG. The sets of confounders we needed to adjust for were (Insurance, Region, Witness, Year, Sex, Place). Therefore, set 1 in the main analysis remains a correct set of variables adjusted for confounding across the modified DAG 1, 2, and 3. Our findings remained valid even if additional links were considered in the original DAG.

## Discussion

The results of this study showed that BCPR significantly increased the chances of survival and good neurological recovery at discharge after adjusting for confounders using IPTW and STR. The effect of BCPR on the outcomes of OHCA did not improve during the study period, although the BCPR rate increased owing to national and regional interventions.

BCPR increased the odds of survival to hospital discharge by 1.67-fold (95% CI: 1.44–1.93). A systematic review in 2018 summarized the effects of BCPR by including 16 cohort studies from Europe (eight studies), North America (four studies), Asia (three studies), and Oceania (one study). A meta-analysis showed that BCPR increased the odds of survival at discharge by 1.95-fold (95% CI: 1.66–2.30). However, substantial heterogeneity was observed (I^2^ = 86.8%), and the pooled odds ratios varied among study locations (2.70 in Oceania, 2.28 in Europe, 1.91 in North America, and 1.36 in Asia). Our study's odds ratios fell between those noted in studies in Asia and North America. The benefit of BCPR could be influenced by confounders, such as age, sex, place of arrest (public vs. private), and witnessed status, in an observational study. However, among the 16 studies, only 10 considered sex, 12 considered witnessed status, and 3 considered the study period. There were no studies that considered age, sex, place, region, witness, insurance, and the study period in the model at the same time. Therefore, a direct comparison of the effect of BCPR on survival to discharge was not possible owing to the different sets of confounders used for adjustment in the models.

Efforts, including dispatch-assisted CPR (DA-CPR), widespread CPR training in the community, and use of a text alert system have been shown to be effective in terms of BCPR provision [23]. In a previous study, a before-and-after design showed that community CPR training and use of a text alert system increased BCPR provision and survival rates in a metropolitan city [24]. Another study on DA-CPR showed improved BCPR provision, survival, and neurological recovery rates in the post-intervention period compared with those in the pre-intervention period [19]. Community CPR training and public CPR campaigns have been conducted as nationwide interventions to disseminate BCPR-related knowledge in Korea [25], and the DA-CPR program has been implemented nationally since 2011 [17]. Hence, the BCPR rate has been steadily increasing from 1.9% in 2008 to 21.0% in 2017, according to KCDC [26]. However, whether this increased rate contributed to the survival and neurological recovery due to improved BCPR quality has not been fully elucidated. The present results showed that the effect of BCPR administered by a layperson on survival and neurological outcomes at hospital discharge did not show a continuously improving trend from 2012 to 2015. According to a review of randomized controlled trials, interventions improving the quality of BCPR include simplified and modified DA-CPR, compression-only CPR, BCPR using a mobile device, and other on-scene interventions such as four-hand CPR for elderly rescuers and heel-CPR for tired rescuers [27]. However, other studies have reported that DA-CPR provision is not correlated with a significant improvement in the survival to discharge rate in a real-world setting and that the current dispatch performance is below the proposed national standards [28]. These findings were consistent with those of another observational study showing negative associations between DA-CPR and outcomes, including ROSC, survival to discharge, and neurological recovery [29]. Furthermore, a review reported conflicting results concerning the survival to discharge between DA-CPR and BCPR [30]. Therefore, the effect of BCPR on the outcomes of OHCA should be monitored using real-world data, to improve the quality of BCPR.

Variations have been reported in the influence of BCPR on survival to discharge and neurological recovery among subgroups. A review showed that the effects of BCPR on survival to discharge differed according to geographical regions (I2 = 86.8%, p <0.001): the odds ratios were 1.91 (95% CI: 1.32–2.77), 2.28 (95% CI: 2.02–2.57), 1.36 (95% CI: 1.16–1.58), and 2.70 (95% CI: 1.13–6.45) in America, Europe, Asia, and Oceania, respectively [11]. However, few studies have focused on the differences in BCPR provision or the effects of BCPR on outcomes according to the characteristics of patients with OHCA. One study reported that women were more likely to receive BCPR and survive to discharge and were less likely to have witnessed OHCA with an initial shockable rhythm [8]. Another study reported that the socioeconomic status of the area where the OHCA event occurred was associated with BCPR provision and survival [7]. Some researchers have proposed that the limited number of studies based on some registries may be because of missing data regarding patient race, ethnicity, and socioeconomic status, limiting the identification of vulnerable populations and evaluating potential disparities in OHCA treatment and outcomes [5]. Furthermore, other studies have suggested that identifying the factors modifying the effects of BCPR is necessary to understand these variations [10]. Therefore, groups with fewer benefits must be evaluated, and modifying factors should be assessed to enhance the effects of BCPR and improve the outcomes of OHCA.

While previous findings regarding the effects of BCPR in certain subgroups are inconsistent, it is well known that the prognosis of patients with OHCA differs among subgroups. Older age, OHCA events at home, initial rhythm other than ventricular fibrillation/tachycardia, and longer duration of no flow are predictors of a poor outcome after OHCA [31]. The subgroup analyses in the present study showed a tendency toward a smaller odds ratio for BCPR administered to patients experiencing OHCA in public places than for BCPR administered to patients experiencing OHCA at home. However, the difference between the odds ratios was not statistically significant. These findings seem to be contrary to the previous studies reporting poor outcomes of OHCA at private places. However, few studies have directly demonstrated a variation in the effect of BCPR on the outcomes of OHCA based on place, while at-home OHCA is well known to have a poorer prognosis than OHCA at public place [32, 33]. A study revealed that BCPR provision and neurologic recovery increased by 2.45- and 1.51-fold in the daytime if friends witnessed OHCA compared with those if family members witnessed OHCA (95% CI: 2.31–2.58, 95% CI: 1.36–1.68) [32]. This suggested that BCPR was provided less in cases of OHCA at home by family members, which may cause poor neurologic recovery; however, it could not suggest the effect of BCPR. The other study showed that the AORs of BCPR without and with dispatcher assist were 1.44 (95% CI: 1.22–1.70) and 1.60 (95% CI: 1.45–1.77) on neurologic recovery, respectively, compared with no BCPR in private places. Additionally, they were comparable to 1.43 (95% CI: 1.24–1.65) and 1.62 (95% CI: 1.43–1.85) in public places [33]. There would be no conflict with the present study even if the BCPR effect was higher in public places in other studies because the outcome estimates in our study were obtained from models considering the interaction between confounding variables, unlike those in other studies. When we eliminated all two-way interactions from the model, the effect of BCPR on survival to discharge and good neurologic recovery was higher for OHCA events occurring at public places than for those occurring at private places (odds ratio = 2.00, 95% CI: 1.73–2.08 for survival to discharge; odds ratio = 1.84, 95% CI: 1.69–2.34 for neurologic recovery). This finding suggested the possible underestimation of the effect of BCPR in OHCA events occurring in private places in previous studies that did not consider the complex relationships between confounders. Further research is needed to validate the effects of BCPR according to place, with more information on the type, provider, and quality of BCPR [34].

In this study, BCPR was beneficial for survival and neurological recovery at discharge, even in patients with an initial non-shockable rhythm or no prehospital ROSC. The initial ECG rhythm was correlated to OHCA prognosis and has been considered the termination of resuscitation by some researchers in combination with other predictors [35]. Most previous studies have shown positive effects of BCPR on the outcomes of OHCA in patients with shockable rhythm at the scene or ROSC before ED arrival. Some studies have reported that prolongation of the shockable phase of ventricular fibrillation due to BCPR may contribute to an increased survival rate to discharge [36]. In contrast, data on the influence of BCPR on the outcomes of OHCA with non-shockable rhythm are lacking and contrasting. Two meta-analyses showed inconsistent relationships between BCPR and survival to discharge in patients with non-shockable rhythm (AORs = 1.85 and 0.62), with substantial heterogeneity [11]. In this study, BCPR was beneficial even in the subgroup of patients with non-shockable rhythm, which might be correlated to the indirect effects of BCPR, such as a later conversion from non-shockable to shockable rhythm or an increase in PEA over asystole [37]. PEA and asystole are commonly considered as monolithic entities. However, a previous study reported that initial PEA had a better prognosis than initial asystole [38, 39].

The current study has several limitations. First, there were missing data regarding BCPR in the national OHCA registry. Information about the provision of BCPR was missing in 74.3% of eligible patients; there were no records of BCPR, although this information was extracted by a trained reviewer from hospital medical records using standardized protocols. While we aimed to evaluate the influence of BCPR on the effect sizes via sensitivity analyses, we would be more likely to overcome this limitation if data merged with EMS prehospital reports were available for research purposes. Second, the causal effect could only be estimated based on the assumption that there were no unobserved confounding factors. However, as the Korea OHCA registry was based on the Utstein 2004 template, some of the elements in the 2015 updates were not available for analysis. Therefore, attention must be paid to a potential bias due to unmeasured confounders when interpreting data. Moreover, DAG was established in this study based on the limited accessible variables, even though a DAG was established based on a review of previous studies and expert opinions. Finally, the study was based on nationwide registry data from Korea. Therefore, the generalizability of our findings may be limited owing to differences in CPR training programs, EMS systems, and patient characteristics.

## Conclusions

This study assessed the effect of BCPR on survival and good neurological outcomes at discharge using data from a nationwide registry. IPTW and STR permitted model interactions among BCPR and confounders and to determine unbiased estimates of the BCPR effect based on DAG. There was a tendency toward a smaller odds ratio for BCPR administered to female patients aged 65 years and older, experiencing OHCA in public places, and with initial non-shockable rhythm. The effect of BCPR on the outcomes of OHCA did not improve during the study period, although the BCPR rate increased owing to national and regional interventions. More efforts are needed to monitor the change in the effect of BCPR on these outcomes to ensure that the benefits of qualified BCPR are evenly distributed and increase over time.

## Supporting information

S1 Table Differences in the characteristics of the study population before and after inverse probability of treatment weighting. NHI, National Health Insurance; IPTW, inverse probability of treatment weighting.
(DOCX)

S2 Table Odds ratios of bystander cardiopulmonary resuscitation for survival and neurological recovery at discharge via standardization. ^a^ The variables in the directed acyclic graph were used as covariates in the model. Set 1: (Place, Insurance, Region, Witness, Year, Sex, Age); Set 2: (Place, Insurance, Region, Witness, Year, Age); Set 3: (Place, Insurance, Region, Witness, Year, Sex). ^b^ Interaction means all the two-way interaction terms among the covariates are included.
(DOCX)

S3 Table Odds ratios of bystander cardiopulmonary resuscitation for survival and neurological recovery at discharge according to patient’s characteristics with out-of-hospital cardiac arrest (OHCA). CPR, Cardiopulmonary resuscitation; OHCA, Out-of-hospital cardiac arrest; NHI, National Health Insurance; Primary rhythm, Primary rhythm at the scene; ROSC, Return of spontaneous circulation.
(DOCX)

S1 Fig Directed acyclic graph for the study outcomes (survival to hospital discharge and good neurological recovery). (TIF)

S2 Fig Graphical comparison of the characteristics of the study population before and after inverse probability of treatment weighting. (TIF)

S3 Fig Odds ratios of bystander cardiopulmonary resuscitation for survival and neurological recovery at discharge according to patient’s characteristics with out-of-hospital cardiac arrest (OHCA) via standardization. (TIF)

S4 Fig Changes in the odds ratios of bystander cardiopulmonary resuscitation (BCPR) for survival and neurological recovery at discharge after considering missing values for BCPR. ^a^ Variables in the directed acyclic graph were used as covariates in the model. Set 1: (Place, Insurance, Region, Witness, Year, Sex, Age); Set 2: (Place, Insurance, Region, Witness, Year, Age); Set 3: (Place, Insurance, Region, Witness, Year, Sex). ^b^ In each plot, “None” means that missing value of BCPR is ignored. “Sensitivity A” refers to the inverse probability weighting for missing BCPR and “Sensitivity B” is similar to “Sensitivity A”, but the outcome variable is also used as covariates in calculating the weight [16].
(TIF)

S5 Fig Sensitivity analysis for the relationships in the directed acyclic graph for outcome (survival to hospital discharge and neurological recovery). (TIF)

S1 Dataset Estimates of OR and 95% CI for survival to discharge and neurological recovery. (DOCX)
