# Supplementary material for: Effectiveness of bystander cardiopulmonary resuscitation in improving the survival and neurological recovery of patients with out-of-hospital cardiac arrest: A nationwide patient cohort study
Source: PLoS One. 2020 Dec 16;15(12):e0243757. doi: 10.1371/journal.pone.0243757 (PMC7744051; doi:10.1371/journal.pone.0243757)
Supplement: S1 Table — NHI, National Health Insurance; IPTW, inverse probability of treatment weighting. (DOCX) [file pone.0243757.s001.docx]

S1 Table. Characteristics of the study population before and after inverse probability of treatment weighting

|  | Standardized difference | |
| --- | --- | --- |
|  | Unweighted | IPTW |
| Age | -0.187 | -0.001 |
| Sex |  |  |
| Male | 0.041 | 0.008 |
| Place |  |  |
| Public | 0.106 | -0.005 |
| Nonpublic | -0.214 | 0.016 |
| Unknown | 0.187 | -0.018 |
| Region |  |  |
| Metropolitan cities | -0.199 | 0.008 |
| Presence of a witness |  |  |
| Witnessed | 0.204 | 0.004 |
| Year |  |  |
| 2012 | -0.319 | 0.012 |
| 2013 | -0.076 | 0.010 |
| 2014 | 0.050 | -0.001 |
| 2015 | 0.298 | -0.017 |
| Insurance |  |  |
| NHI | 0.048 | 0.009 |
| Medical aid | -0.070 | -0.002 |
| Unknown | 0.010 | -0.011 |

NHI, National Health Insurance; IPTW, inverse probability of treatment weighting
