# Supplementary material for: Effectiveness of bystander cardiopulmonary resuscitation in improving the survival and neurological recovery of patients with out-of-hospital cardiac arrest: A nationwide patient cohort study
Source: PLoS One. 2020 Dec 16;15(12):e0243757. doi: 10.1371/journal.pone.0243757 (PMC7744051; doi:10.1371/journal.pone.0243757)
Supplement: S2 Table — a The variables in the directed acyclic graph were used as covariates in the model. Set 1: (Place, Insurance, Region, Witness, Year, Sex, Age); Set 2: (Place, Insurance, Region, Witness, Year, Age); Set 3: (Place, Insurance, Region, Witness, Year, Sex). b Interaction means all the two-way interaction terms among the covariates are included. (DOCX) [file pone.0243757.s002.docx]

S2 Table. Odds ratios of bystander cardiopulmonary resuscitation on survival and neurological recovery via standardization

| Covariates | | Survival to discharge | Neurological recovery |
| --- | --- | --- | --- |
| Set 1^a^ | Interaction^b^ | 1.65(1.44,1.87) | 1.97(1.59,2.36) |
|  | No interaction | 1.65(1.45,1.86) | 1.98(1.62,2.35) |
| Set 2 | Interaction^b^ | 1.65(1.43,1.86) | 1.96(1.58,2.34) |
|  | No interaction | 1.65(1.45,1.86) | 1.99(1.62,2.35) |
| Set 3 | Interaction^b^ | 1.87(1.61,2.12) | 2.34(1.87,2.80) |
|  | No interaction | 1.87(1.63,2.11) | 2.33(1.88,2.78) |

^a^ The variables in the directed acyclic graph were used as covariates in the model.

Set 1 : (Place, Insurance, Region, Witness, Year, Sex, Age);
Set 2 : (Place, Insurance, Region, Witness, Year, Age);
Set 3 : (Place, Insurance, Region, Witness, Year, Sex)

^b^ Interaction means all the two-way interaction terms among the covariates are included.
