# Supplementary material for: Effectiveness of bystander cardiopulmonary resuscitation in improving the survival and neurological recovery of patients with out-of-hospital cardiac arrest: A nationwide patient cohort study
Source: PLoS One. 2020 Dec 16;15(12):e0243757. doi: 10.1371/journal.pone.0243757 (PMC7744051; doi:10.1371/journal.pone.0243757)
Supplement: S3 Table — CPR, Cardiopulmonary resuscitation; OHCA, Out-of-hospital cardiac arrest; NHI, National Health Insurance; Primary rhythm, Primary rhythm at the scene; ROSC, Return of spontaneous circulation. (DOCX) [file pone.0243757.s003.docx]

S3 Table. Odds ratios of bystander CPR on survival and neurological recovery according to the characteristics of the patients with OHCA

|  |  | Survival to discharge | | Good neurological recovery | |
| --- | --- | --- | --- | --- | --- |
|  |  | IPTW | STR | IPTW | STR |
| Age | < 65 years | 1.94  (1.61,2.35) | 1.98  (1.64,2.32) | 2.04  (1.60,2.61) | 2.11  (1.64,2.58) |
|  | ≥ 65 years | 1.37  (1.10,1.72) | 1.39  (1.09,1.69) | 2.12  (1.34,3.34) | 2.16  (1.24,3.07) |
| Sex | Male | 1.84  (1.55,2.18) | 1.84  (1.57,2.11) | 2.26  (1.77,2.88) | 2.28  (1.79,2.76) |
|  | Female | 1.29  (0.98,1.70) | 1.26  (0.95,1.56) | 1.19  (0.76,1.88) | 1.22  (0.76,1.69) |
| Place | Public | 1.42  (1.12,1.80) | 1.39  (1.09,1.68) | 1.62  (1.18,2.22) | 1.64  (1.18,2.12) |
|  | Private | 2.07  (1.71,2.51) | 1.92  (1.58,2.26) | 3.00  (2.17,4.15) | 2.57  (1.83,3.32) |
| Insurance | NHI | 1.77  (1.52,2.07) | 1.76  (1.52,1.99) | 2.10  (1.67,2.65) | 2.13  (1.71,2.55) |
|  | Medical aid | 1.40  (0.84,2.33) | 1.21  (0.63,1.79) | 1.50  (0.65,3.49) | 1.19  (0.31,2.08) |
| Region | Metropolitan | 1.55  (1.30,1.84) | 1.55  (1.32,1.79) | 1.81  (1.40,2.33) | 1.84  (1.44,2.24) |
|  | Non-metropolitan | 1.89  (1.47,2.42) | 1.82  (1.42,2.22) | 2.29  (1.54,3.41) | 2.30  (1.50,3.11) |
| Witness | Witnessed | 1.68  (1.41,2.00) | 1.84  (1.43,2.25) | 1.98  (1.54,2.55) | 2.33  (1.51,3.15) |
|  | Unwitnessed | 1.60  (1.22,2.09) | 1.62  (1.21,2.04) | 1.64  (1.03,2.63) | 1.85  (1.02,2.69) |
| ROSC | Recovery | 2.09  (1.25,3.50) | 2.20  (1.16,3.23) | 1.60  (1.00,2.57) | 1.47  (0.86,2.09) |
|  | Non-recovery | 1.19  (1.00,1.41) | 1.19  (1.00,1.38) | 1.13  (0.80,1.59) | 1.17  (0.80,1.55) |
| Primary rhythm | Shockable | 1.85  (1.46,2.33) | 1.84  (1.45,2.22) | 1.94  (1.49,2.53) | 1.86  (1.42,2.31) |
|  | Non shockable | 1.47  (1.21,1.79) | 1.44  (1.18,1.70) | 1.83  (1.18,2.85) | 1.99  (1.21,2.77) |
| Year | 2012 | 1.78  (1.34,2.37) | 1.77  (1.31,2.23) | 1.65  (1.08,2.53) | 1.62  (0.99,2.25) |
|  | 2013 | 1.79  (1.34,2.41) | 1.80  (1.32,2.28) | 2.02  (1.29,3.07) | 2.16  (1.29,3.04) |
|  | 2014 | 1.66  (1.29,2.13) | 1.52  (1.17,1.86) | 2.43  (1.65,3.56) | 2.23  (1.44,3.03) |
|  | 2015 | 1.68  (1.27,2.21) | 1.60  (1.21,1.99) | 1.91  (1.29,2.83) | 1.89  (1.24,2.55) |

CPR, Cardiopulmonary resuscitation; OHCA, Out-of-hospital cardiac arrest; NHI, National Health Insurance; Primary rhythm, Primary rhythm at the scene; ROSC, Return of spontaneous circulation
