# Supplementary figures and images for: Effectiveness of bystander cardiopulmonary resuscitation in improving the survival and neurological recovery of patients with out-of-hospital cardiac arrest: A nationwide patient cohort study
Source: PLoS One. 2020 Dec 16;15(12):e0243757. doi: 10.1371/journal.pone.0243757 (PMC7744051; doi:10.1371/journal.pone.0243757)

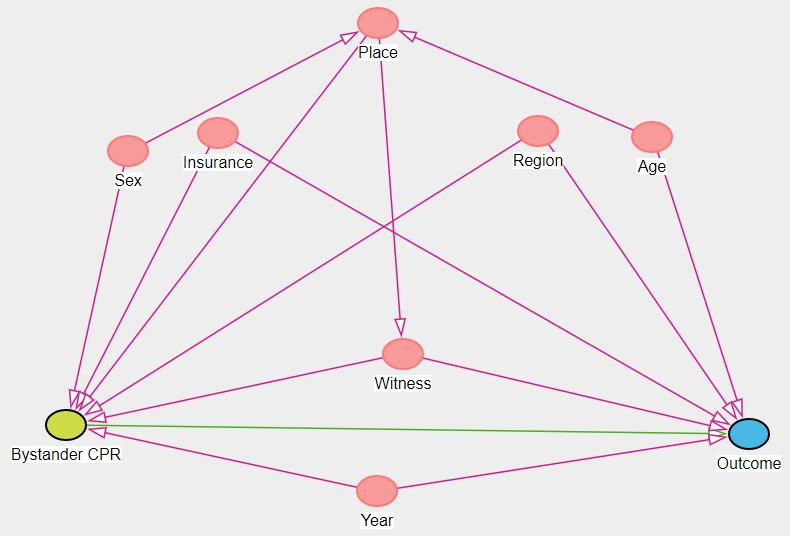

Supplement: S1 Fig — (TIF) [file pone.0243757.s004.tif]

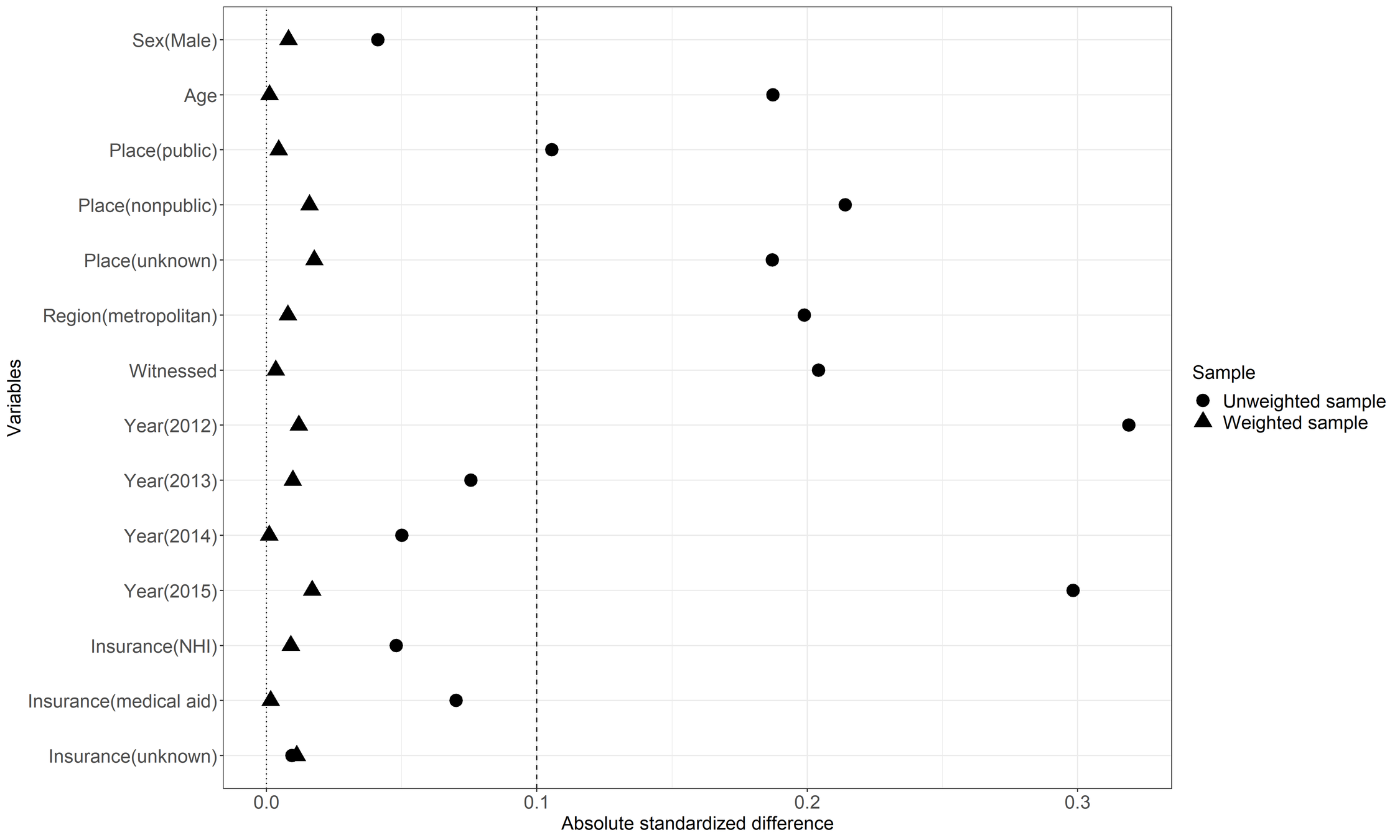

Supplement: S2 Fig — (TIF) [file pone.0243757.s005.tif]

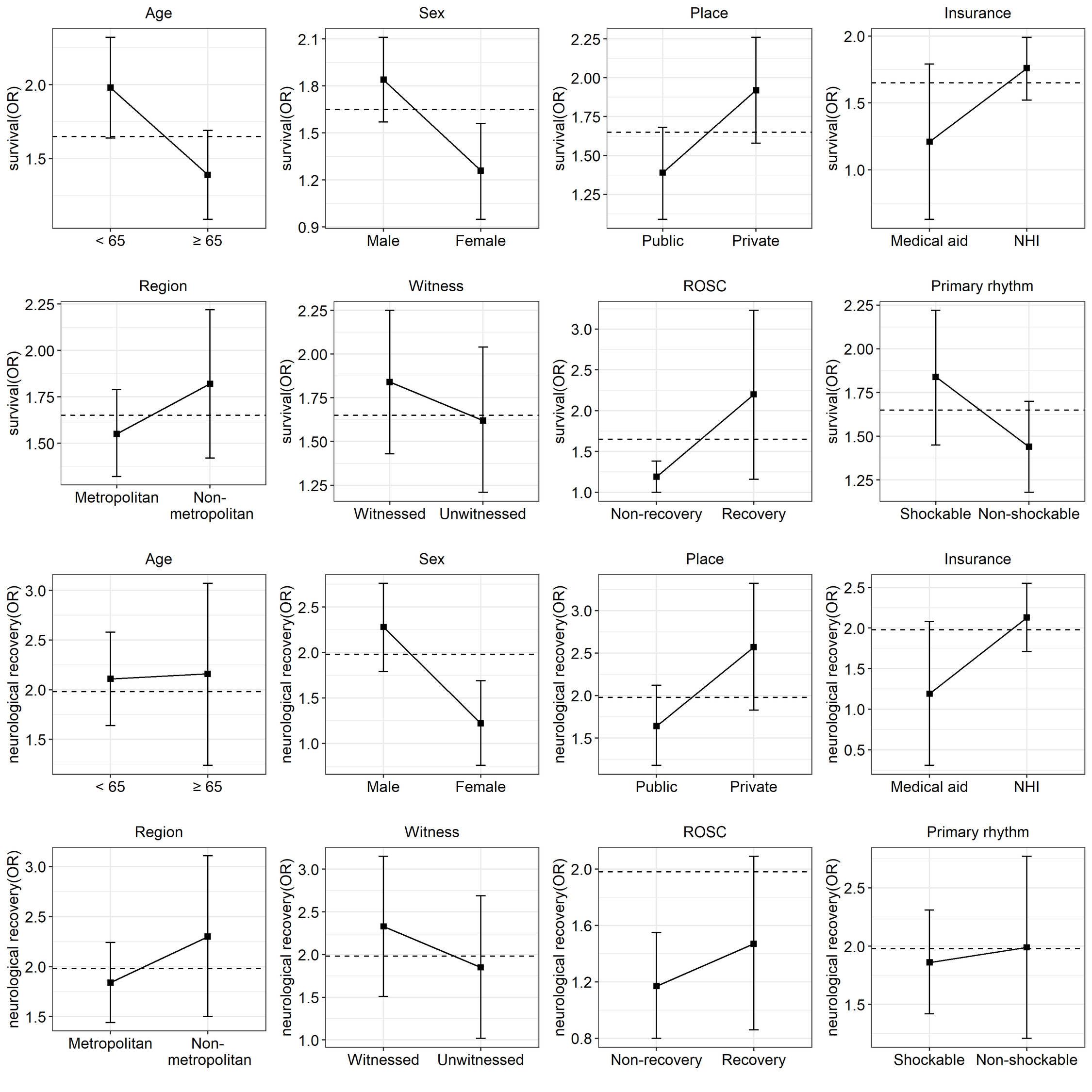

Supplement: S3 Fig — (TIF) [file pone.0243757.s006.tif]

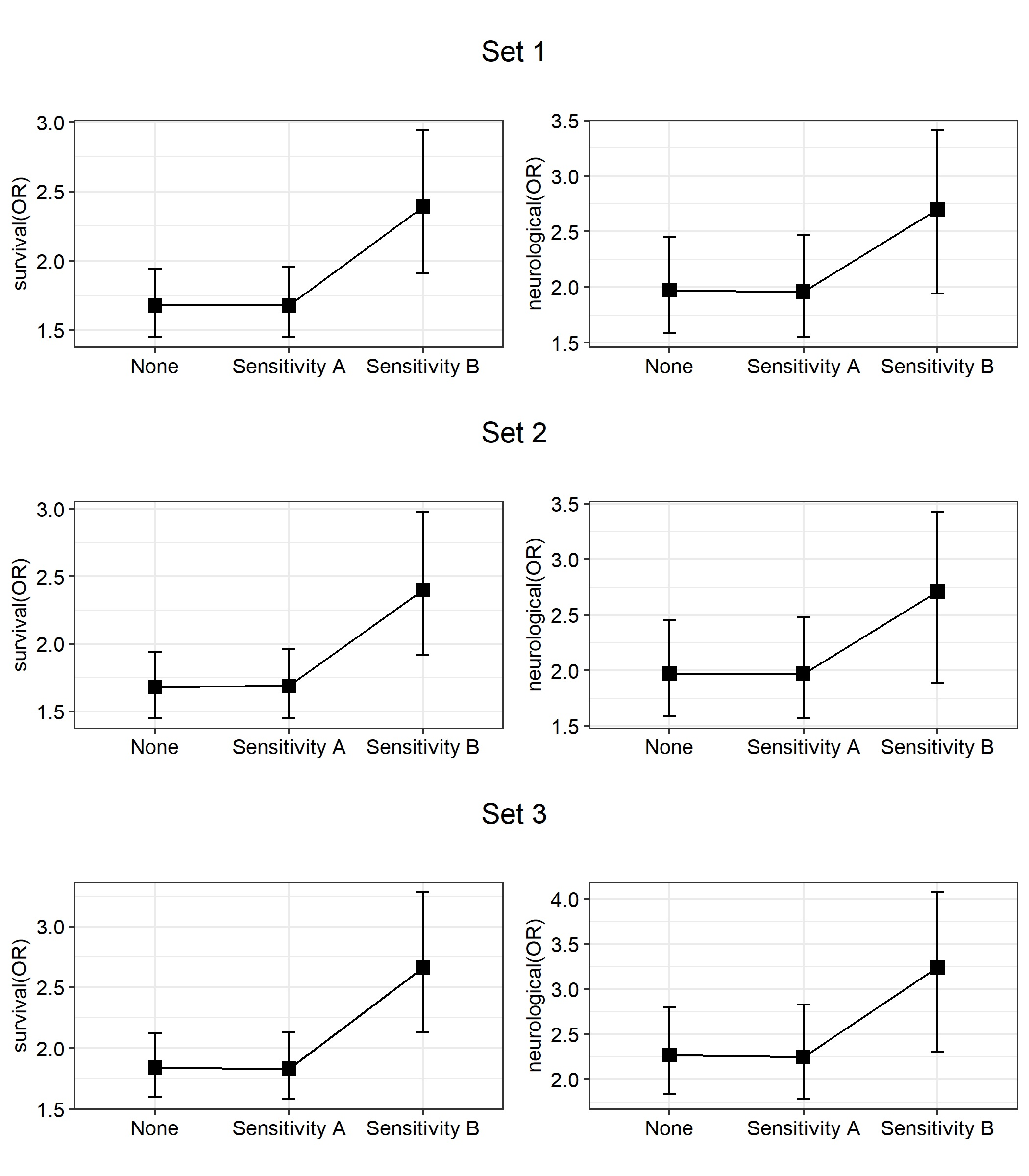

Supplement: S4 Fig — a Variables in the directed acyclic graph were used as covariates in the model. Set 1: (Place, Insurance, Region, Witness, Year, Sex, Age); Set 2: (Place, Insurance, Region, Witness, Year, Age); Set 3: (Place, Insurance, Region, Witness, Year, Sex). b In each plot, “None” means that missing value of BCPR is ignored. “Sensitivity A” refers to the inverse probability weighting for missing BCPR and “Sensitivity B” is similar to “Sensitivity A”, but the outcome variable is also used as covariates in calculating the weight [16]. (TIF) [file pone.0243757.s007.tif]

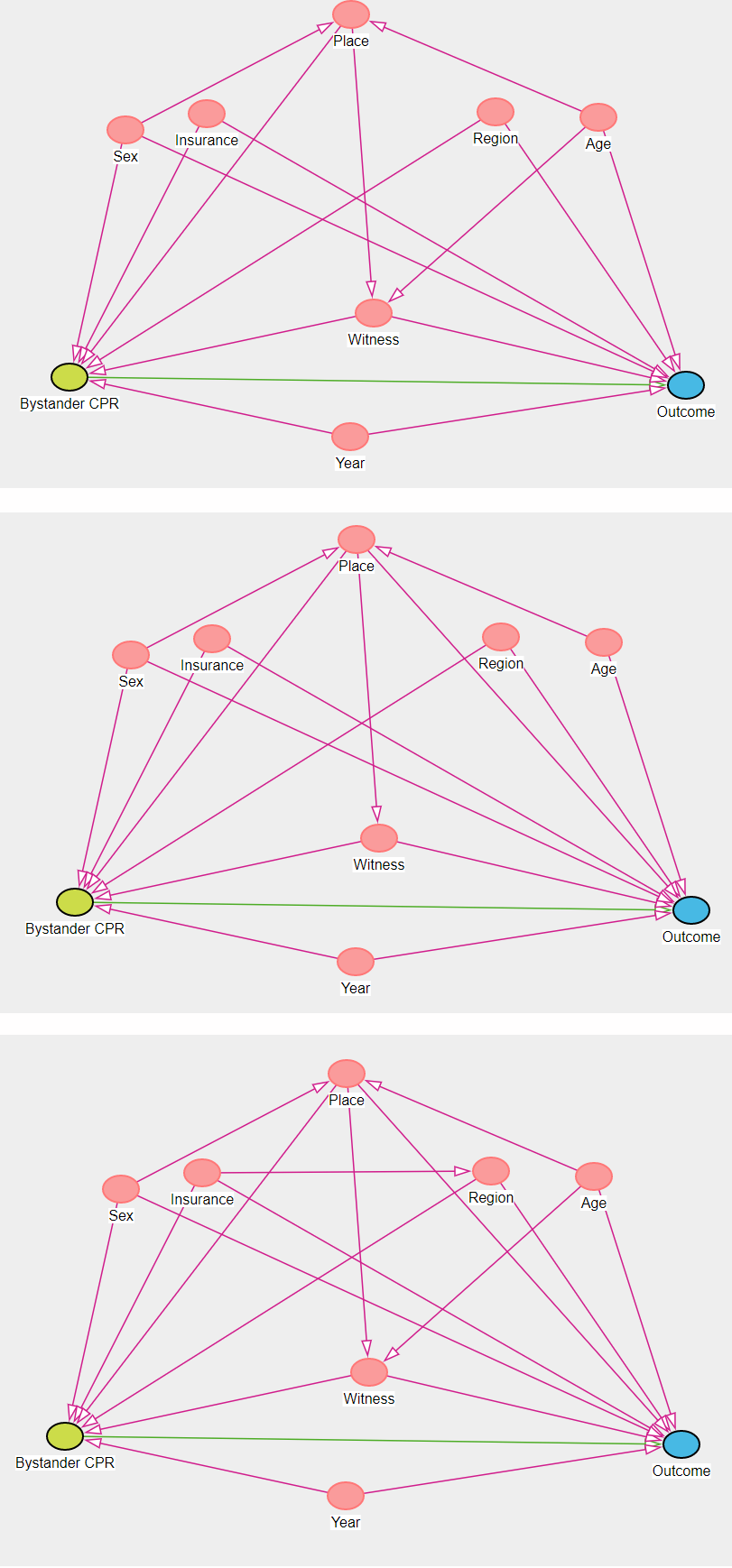

Supplement: S5 Fig — (TIF) [file pone.0243757.s008.tif]
